# Short-Term Storage and Executive Working Memory Processing Predict Fluid Intelligence in Primary School Children

**DOI:** 10.3390/jintelligence5020017

**Published:** 2017-04-28

**Authors:** Eva A. Aeschlimann, Annik E. Voelke, Claudia M. Roebers

**Affiliations:** Department of Psychology, University of Bern, 3012 Bern, Switzerland; avoelke@gmx.ch (A.E.V.); claudia.roebers@psy.unibe.ch (C.M.R.)

**Keywords:** intelligence, fluid intelligence, verbal and visual-spatial working memory, executive processing, short-term storage, children, cognitive development

## Abstract

Working memory (WM) includes short-term storage and executive processing of information. WM has been suggested to be one of the key concepts to explain individual differences in fluid intelligence (Gf). However, only a few studies have investigated the association of the two different aspects of WM in relation to Gf. Furthermore, even fewer studies have included children. Therefore, we first investigated the inter-relations between the WM aspects (verbal and visual-spatial storage, verbal and visual-spatial executive processing). Second, we explored the relation between a general WM factor and Gf. Third, we analyzed the relations between the different WM aspects and Gf while we controlled for common variance among all WM tasks. Nine- to 11-year olds had to solve simple and complex span tasks. Correlations and structural equation modeling techniques were used to examine these relations. Most inter-relations among simple and complex spans were found to be substantial and positive. The general WM factor was related to Gf. Furthermore, after controlling for common variance among all WM tasks, individual differences in verbal storage, visual-spatial storage and verbal processing still uniquely related to Gf. Visual-spatial processing, however, was not related to Gf. Results are discussed in terms of underlying mechanisms.

## 1. Introduction

Children and adults performing better on intelligence tests are typically also more successful in school- and work-related settings and live healthier and longer [1]. Because of its importance in many domains, researchers have been interested in the study of intelligence for more than a century. One topic that has been addressed is cognitive correlates of intelligence such as processing speed, attention, inhibition and working memory (WM). This is done to shed light on the underlying information processes of intelligence, both in children and in adults, as intelligence is a very broad construct [2,3,4,5,6,7,8].

WM has been investigated intensively in relation to intelligence, regarding its relation with fluid intelligence (Gf). Previous studies consistently report that higher WM performance is associated with higher intelligence [9,10,11,12]. The reasons for this substantial relation, however, are still being discussed. A better understanding of the underlying information processes contributing to the WM–intelligence link appears to be of special significance in the context of cognitive development. On the one hand, this is because WM is not only related to intelligence but also to cognitive performance in many domains, including school achievement. On the other hand, WM is also found to contribute to growth in various areas of cognitive development such as language, reading and mathematics [13,14,15,16,17]. Against this background, understanding the information processes involved in the WM–intelligence link in children seems to be of great theoretical and practical importance.

WM is a complex theoretical construct. While conceptualizations of WM differ strongly, most researchers agree that WM contains a short-term storage aspect and an executive processing aspect [18,19]. The distinction between these two aspects of WM may in fact be crucial for understanding the WM–intelligence relation. Unfortunately though, this has only rarely been addressed and existing results are inconsistent [12]. While some studies suggest that the individual’s capacity to store a certain amount of information for a short time drives the relation to intelligence, other studies found a predominance of executive processing in the WM–intelligence link [8,12,20]. Especially in young samples, evidence concerning the specific contribution of short-term storage and executive processing is scattered and inconsistent, a fact that constitutes the starting point for the present approach. We will explore the relations among WM short-term storage, WM executive processing and Gf in a sample of elementary school children.

### 1.1. Definition of Key Concepts

In the present work, we focused on *fluid intelligence* (Gf). Gf is a complex ability that allows us to adapt our thinking to a new cognitive problem or situation [21]. Compared to other intelligence constructs, Gf is sought widely independent of experience and unrelated to culture and language [22,23]. Furthermore, Gf explains around 80% of variance in general intelligence in children [23]. Typically, Gf is measured with tests in which individuals are confronted with figural problems. To solve such problems, inductive and deductive reasoning is needed [23].

There are different theories that vary in their definitions of *WM*. Despite this heterogeneity, what all the theories have in common is that they provide a description of how individuals temporarily store information during cognitive processing. Moreover, experts agree that WM is a capacity-limited system. Hence, within this system, only a fair amount of information can be maintained during a very limited period of time [19,24,25,26,27]. Thus, WM comprises different aspects of information processing and is viewed as a multifaceted construct with interacting *storage* and *executive processing* [28,29,30].

The *storage* aspect describes the maximum amount of information an individual can possibly store for a short time. It is also called short-term memory or short-term memory capacity and is required when a small amount of information must be held in an active state. Short-term storage facilitates the processing of task relevant information [6,28] and has been found to be of special importance for first and second language learning [31].

The *executive processing aspect* is much more difficult to define as it is heterogeneously denoted by different researchers, including terms like “control of attention”, “executive control”, “cognitive control”, “controlled processing” or “executive attention” [6,8,25]. As an exhaustive review of these denotations is beyond the scope of the present paper, we focus on the most typical operationalization. Namely, executive processing is defined as the residual variance left in WM after variance of storage has been controlled for. In other words, executive processing subsumes all the mental processes that are left when storage is held constant. Thus, this residual executive processing variance contains mental operations that go beyond passive storage, including attention and cognitive control processes [6,25]. In the present work, when using the term *WM*, we refer to the whole WM system including storage and executive processing aspects. With the term *storage*, we refer to the short-term storage aspect. With the term *processing*, we refer to the executive processing aspect.

### 1.2. Measurement Challenges in WM

Storage and processing are measured by means of differing tasks. To assess the storage aspect, so-called *simple span tasks* are typically used. For example, an individual is presented with a sequence of digits and is asked to recall them in the same order as presented after a minimal delay. Thus, to solve a simple span task, mainly short-term storage is required. To assess the processing aspect of WM, so-called *complex span tasks* are typically used [32]. In these tasks, individuals are asked to keep some information in mind while simultaneously processing the same or additional information [18,33]. For example, an individual is presented with a sequence of digits and is asked to recall them in reversed order after a minimal delay. Thus, the executive processing aspect of WM can only be assessed indirectly because complex span tasks also trigger short-term memory processes. Hence, it is difficult to interpret WM task results [34].

As simple and complex span tasks both contain the storage aspect, it is not surprising that typically they substantially correlate. When aiming to establish the involved information processes, that contribute to the WM–intelligence link, in isolation (i.e., storage and processing), high correlation among constructs leads to the problem of multicollinearity [20,35]. It implies that the unique contributions of the single predictors are likely to be either under- or overestimated, because, shared variances are attributed to either one of the included inter-correlated predictors. Typically, one way to solve this problem is to control for storage variance in complex span tasks and thereby capture the “pure” processing variance [20]. It is important to note that the problem of multicollinearity does not only occur because complex span tasks demand storage. It additionally occurs because simple span tasks also demand executive processing. Specifically, visual-spatial simple span tasks demand executive processing [36,37]. Such findings also challenge the classical measurement of the storage aspect of WM and implicate that common variance between simple and complex span tasks may differ between the verbal and visual-spatial modality. In fact, in the verbal modality, common variance seems to be storage. In contrast, for the visual-spatial modality, evidence is rare but suggests that, besides storage, common variance is also mirrored in the processing aspect of WM [33,36,37]. 

In the next sections, we will discuss previous studies examining the relation between WM and Gf. Note that, in all discussed studies, authors controlled for common variance among simple and complex span tasks. They controlled for common variance either with the use of hierarchical regression analyses or by using structural equation modeling techniques (SEM). 

### 1.3. Relationship between WM and Gf

In literature, it has been emphasized that WM is the concept that best predicts individual differences in Gf [38,39]. Some authors have even suggested that WM and Gf represent the same construct [40,41]. However, most experts consider them separable constructs, both in adults and children [8,9,10,12,42,43]. Meta-analyses with adult data estimated the correlation between WM and Gf to vary between *r* = .72 and *r* = .85 [9,10]. In recent studies with children, WM has been found to correlate with Gf as high as *r* = .77 [44]. 

Given this strong overlap, is it the storage or processing aspect that is the basic mechanism driving the relation between WM and Gf? Inconsistent results have been reported so far. Studies with adults revealed that the relation between WM and Gf is mainly driven by executive processing [25,45], while more recent research suggested that storage also explains substantial amounts of variance in Gf [20,28,46,47].

Studies including children produced a mixed pattern of results: while some findings suggest that only processing is a predictor of Gf [6,7,48], other findings indicate that storage and processing are equally strong predictors [49]. Further findings indicate that either storage or processing is the stronger predictor of Gf [4,8,12,50]. In summary, existing findings suggest that processing is certainly one substantial mechanism underlying the connection between WM and Gf. For storage, however, findings are inconsistent. Explanations for these different results vary strongly, including the nature of the WM system itself, the analyzed samples, developmental changes as well as the modality (verbal or visual-spatial) of the included tasks [8,12,18,35].

In fact, an important issue within the literature on the WM–intelligence link concerns the modality of the to-be-recalled material [18,35,49]. In other words, the question arises whether processing and storage are both related to Gf when analyzed separately for the verbal and visual-spatial modality. This is essential because it provides a more detailed understanding of the mechanisms underlying the connections between WM and Gf [43]. Especially for storage, findings suggest that it should be analyzed separately for the verbal and visual-spatial modality. For example, results from individual differences approaches with children point out that storage is modality-specific [30]. Further studies analyzing individuals with atypical development revealed selective deficits for the verbal storage aspect of WM, but not for the visual-spatial storage aspect [51]. For the processing aspect, in contrast, previous studies suggest that the link to intelligence is likely to generalize for both the verbal and the visual-spatial modality [33,43]. Taken together, these findings imply that individual differences in WM have diverse sources that may all contribute to the WM–intelligence link. However, only a few studies analyzed the relation between storage and processing and Gf separately for the verbal and visual-spatial modality. In the next section, findings of these studies including children will be discussed.

Studies investigating the predictive power of the WM processing aspect for the verbal modality indicate that verbal processing, in fact, predicts significant amounts of variance in Gf [7,8,48,50]. However, to the best of our knowledge, only one study explored the relation between the WM processing aspect and Gf separately for the verbal and visual-spatial modality [49]. The authors found that verbal and visual-spatial processing are equally important for Gf. This result is in line with findings showing that WM processing is modality-general [33,43]. In summary, findings indicate that verbal processing predicts Gf. However, because of the small number of studies, the question whether visual-spatial processing predicts Gf over and beyond verbal processing is yet to be thoroughly investigated, especially in young samples.

Studies investigating the predictive power of the WM storage aspect for the verbal modality indicate that verbal storage does not predict variance in Gf [6,7,48]. In contrast, the few studies that explored the relation between the WM storage aspect and Gf separately for the verbal and visual-spatial modality indicate conflicting results. Namely, two studies found that verbal and visual-spatial storage both predict variance in Gf [49,50], whereas another study found that only visual-spatial storage was related to Gf, but not verbal storage [52]. Together, these findings indicate that visual-spatial storage predicts Gf. However, the question whether verbal storage predicts Gf over and beyond visual-spatial storage in children remains open.

### 1.4. The Present Study

The main aim of the present work was to investigate the relations between the four WM aspects (verbal storage, visual-spatial storage, verbal processing and visual-spatial processing) and Gf. For this purpose, the storage and processing aspect of WM were measured with tasks that were—within their modality—as similar as possible, and differed only with respect to the additional processing demand. Due to the problem that simple and complex span tasks always share variance, we firstly analyzed the inter-relations among these tasks [20]. Secondly, we studied the relation between a general WM factor and Gf. Thirdly, relations between the different WM aspects and Gf were examined. To calculate relations among WM (respectively, WM aspects) and Gf, we used SEM, hence taking the inter-relations among the storage and processing tasks and among the modalities into account. The method of SEM has different advantages. Particularly, it enables controlling for common variance of the simple and complex span tasks when exploring the relations between different WM aspects and Gf. 

To get a more detailed understanding of the mechanisms underlying the connections between WM and Gf, we studied the relations between Gf and the WM aspects separately for both the verbal and visual-spatial modality. To the best of our knowledge, there is only one study that investigated the connection between storage, processing and Gf separately for verbal and visual-spatial tasks [49]. Especially for young samples, our study will thus make a unique contribution to illuminating the WM information processes involved in Gf.

As for the present work, simple and complex span tasks were very similar within the modality (i.e., verbal vs. visual-spatial). However, across modalities, they differed greatly, hence why we assumed a stronger link between the two tasks within one modality than between the two tasks of comparable complexity. Furthermore, we examined whether the visual-spatial simple span task would not only demand the storage aspect of WM, but also the executive processing aspect of WM, something that has been suggested by very few previous studies.

Concerning the WM–intelligence link, we predicted that the general WM factor would explain substantial variance in Gf. When analyzing the relations between the different WM aspects and Gf, we assumed the following: verbal processing predicts unique variance in Gf, over and beyond the common variance that is captured by the WM factor. Because only few studies explored the relation between visual-spatial processing and Gf, we had no firm hypothesis as to visual-spatial processing predicting variance in Gf. As for the storage aspects, previous research is also rare, and our study is explorative in nature. For both verbal and visual-spatial storage, we aimed to explore whether storage explains significant amounts of variance in intelligence, over and beyond the processing aspects.

## 2. Materials and Methods

### 2.1. Participants

The sample consisted of 127 children between the age of 9 (*N* = 57; 51% girls; mean age = 114 months; *SD* = 4; age range: 108–122 months) and 11 years (*N* = 70; 49% girls; mean age = 138 months; *SD* = 3; age range: 132–144 months). Participants were recruited from public schools in the vicinity of a University town. In addition, 69% of the children had Swiss German or German as the first language, 25% were bilingual with Swiss German or German and a second language as the first language. The remaining children had sufficient German language skills as to understand the instructions. Written consent was obtained from the main caregiver of the participating children; children gave oral consent. 

The study was conducted in accordance with the Declaration of Helsinki, and the protocol was approved by the local ethics committee of the faculty (project identification code: 2011-06-103). The intelligence quotient (IQ) was normally distributed in the sample (skewness = −0.22; kurtosis = −0.48) with a mean only slightly above 100 (*M* = 102.39; *SD* = 12.15; range = 75–128). This is important to note because homogenous IQ samples are not supposed to show the same relations between Gf and WM as normally distributed samples do [35].

### 2.2. Tasks

#### 2.2.1. Assessment of Gf

Fluid intelligence (Gf) was assessed with the short version of the German adaptation of Cattel’s Culture Fair Test (CFT 20-R; reliability of.92; [53]). The CFT 20-R is a paper–pencil task and consists of four subtests (Series Completion, Classification, Matrix Completion, and Topological Reasoning). All subtests have a time limit. For the descriptive statistics and the correlations, we used the sum of correct answers across all four subtests as dependent variable of Gf. For the SEM, the sum of correct answers for each subtest was used separately. 

#### 2.2.2. Assessment of WM Aspects (Simple and Complex Span Tasks)

An overview of the span tasks used to measure the WM aspects is shown in Table 1. To control for storage in a complex span task, we applied tasks in which the same information per modality had to be stored across different levels of complexity (digits in the verbal tasks and blackened squares in the visual-spatial tasks). For measures of storage, we included forward versions; for measures of executive processing, backward versions were used. The forward versions represent simple span tasks. Because the sequences are immediately reproduced, the processing load is assumed to be minimal. In contrast, for reproducing the sequences backwards (i.e., complex span tasks), additional executive processing is needed [25,33,54].

In the present study, span tasks consisted of six trials per block and sequence length, respectively. Each task started with a training block of four trials. Note that a training trial was repeated if recall was inaccurate. After the training block, each participant started with the length of two digits or two blackened squares, respectively. A trial was considered correct when all digits or squares were reproduced in the correct order. With four correct trials out of the six trials within one sequence length, the next block was administered, including trials of one additional digit or blackened square, respectively. If less than four trials were reproduced correctly, the task was terminated. Before a new block started, children were informed about the length of the next trials. While instructions were given orally, stimuli were presented computer-based in order to increase standardization and to attain a higher reliability (Computer: Acer W700 with a 26.0 cm × 14.4 cm and 1920 × 1080 pixel touch-screen; Software: E-Prime [57,58]). 

In the verbal span tasks, digits were played by headphones (Sennheiser HD 201, Wedemark, Germany) at a rate of one digit per second. Children had to respond orally after the last digit and were thereby being protocolled by the experimenter.

In the visual-spatial tasks, blackened squares were presented in a 4 × 4 matrix (size of the matrix: 13.2 cm × 13.2 cm; size of each field within the matrix: 3.3 cm × 3.3 cm). Every blackened square appeared for 1.2 s and disappeared when the next blackened square showed up. Every last blackened square was first followed by a screen with an interrogation mark for 1 s and then by an empty 4 × 4 matrix. Children had to type their answers directly into this empty matrix on the touch-screen, with the computer recording the answers. When finished, children were asked to put back their hand onto a pad in front of the computer.

For all of the span tasks, there was no time limit. Hence, the next trial started whenever the child indicated to be ready. For each span task, the dependent variable was the total number of correctly answered trials.

### 2.3. Procedure

During normal school hours, participants solved the tasks in two sessions. In one of these sessions, participants solved the CFT 20-R task in small groups of four to seven children (duration: max. 45 min). In the other session, participants completed the span tasks individually in a separate and quiet room with their experimenter (duration: max. 40 min). Every child started with the simple span tasks and ended with the complex span tasks, while verbal and visual-spatial tasks alternated. The starting modality was counterbalanced among the children. At the end of the last session, participants received a small gift.

### 2.4. Data Analysis

Data was analyzed using the software SPSS statistics 23 and Amos 23 [59,60]. Partial correlations were used to examine the inter-relations among the simple and complex span tasks, and SEM was used to examine the relation among the WM aspects and Gf. For the SEM, fits were considered good if the chi-square probability was greater than .05, the normed χ^2^ was below 2, the comparative fit index (CFI) was greater than .95, the root mean square error of approximation (RMSEA) was smaller or equal to .06, and the standardized root mean square residual (SRMR) was smaller than .10 [61,62].

## 3. Results

Results are organized in three sections. Firstly, we provided descriptive statistics and compared performances in the four span tasks. Secondly, we examined the inter-relations among the simple and complex span tasks. Thirdly, we investigated the relation between WM and Gf. For this, we (a) analyzed the prediction of one general WM factor onto Gf; and (b) we explored the prediction of each WM aspect onto Gf.

### 3.1. Descriptive Statistics

Descriptive statistics of performance level of simple and complex span tasks and Gf are displayed in Table 2. Kurtosis and skewness were within the range of ±1.0. Thus, data may be assumed to be normally distributed [61].

To get a better understanding of the simple and complex span tasks, we analyzed performance differences between them. Results of the analysis of variance revealed that children achieved higher scores in the forward tasks (simple span tasks) compared to the backward tasks (complex span tasks), *F*(1,126) = 307.51, *p* < .001, and η*_p_*^2^ = .71. Furthermore, children achieved higher scores in the verbal span tasks compared to the visual-spatial span tasks, *F*(1,126) = 36.54, *p* < .001, and η*_p_*^2^ = .22. It is important to note that the interaction between the modality (verbal vs. visual-spatial), and the order/complexity (forward vs. backward) was also significant: in both modalities, children achieved higher scores in the simple span tasks (forward versions) compared to the more complex ones. However, the effect was greater for the verbal simple span than for the visual-spatial simple span, *F*(1,126) = 131.35, *p* < .001, and η*_p_*^2^ = .51. 

Pearson correlations (Table 3) showed that all tasks are positively related to each other. Age correlated positively with all variables from *r* = .22 until *r* = .45. Previous studies found that storage and processing predict Gf differently depending on age [8,12,63]. Furthermore, previous studies found that age is related to storage, processing and/or Gf [4,6,12,34,64,65]. Because of these reasons, we controlled for age in all model analysis and computed additional models to examine the predictive power of age onto Gf. For further analysis, all variables were *z*-standardized.

### 3.2. Inter-Relations between Simple and Complex Span Tasks

**Figure 1:**
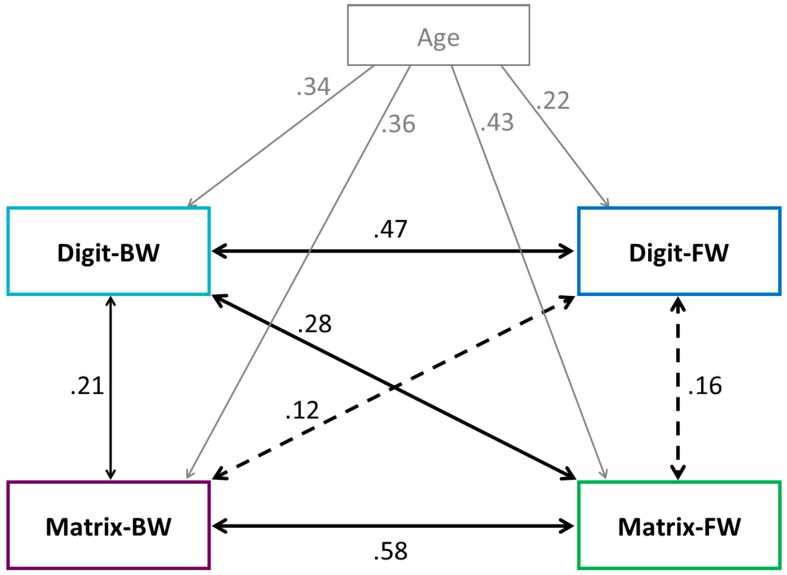
Correlations between age and the four WM aspects as well as partial correlations after controlling for age between the four WM aspects. Digit-FW = digit forward; Digit-BW = digit backward; Matrix-FW = matrix forward; and Matrix-BW = matrix backward. *Solid lines* represent significant correlations (*p* < .05), *dashed lines* represent non-significant correlations (*p* > .05).

To analyze inter-relations among the WM aspects, partial correlations were calculated with age as control variable (see Table 3 or Figure 1 with an illustration of the correlations). Results showed that all correlations were significant and positive (all *p* < .05), except for two; namely, the correlations between the verbal simple span and the two visual-spatial spans. As expected, correlations among modalities (verbal vs. visual-spatial) were stronger (correlation between simple and complex spans within the modalities: verbal *r* = .47; visual-spatial *r* = .58) than among task complexities (simple vs. complex; correlation between the complex spans: *r* = .21; correlation between the simple spans: *r* = .16, non-significant).

Next, we explored whether the visual-spatial simple span task not only demanded the storage aspect of WM, but also the executive processing aspect of WM. Hence, we looked at the correlations between the visual-spatial simple span task and the two complex span tasks. Results revealed that visual-spatial simple span correlated with visual-spatial complex span (*r* = .58). Additionally, visual-spatial simple span correlated with verbal complex span (*r* = .28). Surprisingly, the strength of these correlations was moderate to large [66]. Consequently, we can assume that visual-spatial simple span demands not only storage, but also processing. This indicates that, above storage, shared variance between visual-spatial simple span and visual-spatial complex span mirrors mainly processing.

### 3.3. Relationship between WM and Gf

A SEM was performed to investigate first the relation among a common WM factor and Gf, and second, to investigate the relations between the different WM aspects and Gf. We used a two-step modeling approach. In the first step, we tested the measurement model. After a good fit of the measurement model was confirmed, the second step of testing the structural model followed [61,62].

**Figure 2:**
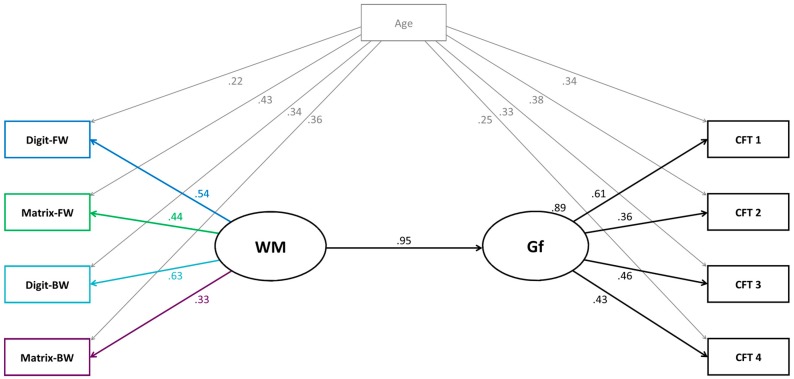
Structural equation model (Model 1a) testing the relation between one working memory (WM) factor and fluid intelligence (Gf). Age (in months) was included as control variable. Digit-FW = digit forward; Digit-BW = digit backward; Matrix-FW = matrix forward; Matrix-BW = matrix backward; CFT 1–4 = subtests of the CFT 20-R. All paths were significant (*p* < .05).

The measurement model was built as follows: all four WM tasks were to load on one WM factor, and all Gf tasks loaded on one Gf factor. The WM factor correlated with the Gf factor. Additionally, we controlled for age by regressing each WM task and each Gf task onto age. The model generated a good fit [*χ*^2^ (18) = 16.63, *p* = .55, normed *χ*^2^ = 0.92; CFI = 1.00; RMSEA = .00; SRMR = .04]. Next, we built the SEM (Model 1a). This model was built as the measurement model, except that Gf was regressed onto WM. See Figure 2 with the results of this model. The model generated an excellent fit to the data and explained 79% of the variance in Gf [*χ*^2^ (18) = 16.63, *p* = .55, normed *χ*^2^ = .92; CFI = 1.00; RMSEA = .00; SRMR = .04]. All regression coefficients of the model were significant at *p* < .05.

**Figure A1:**
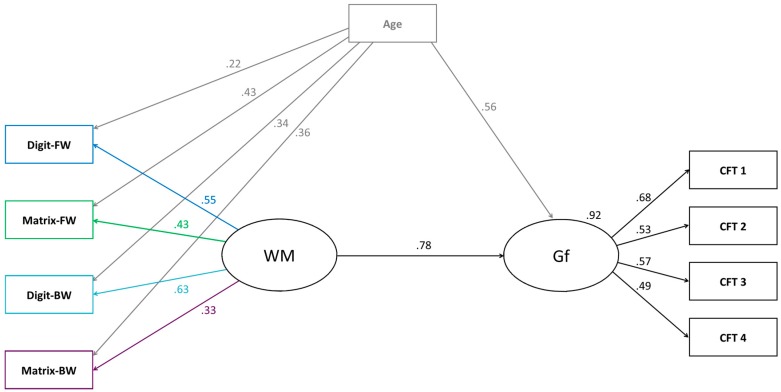
Structural equation model (Model 1b) testing the relation between one working memory (WM) factor, age and fluid intelligence (Gf). Digit-FW = digit forward; Digit-BW = digit backward; Matrix-FW = matrix forward; Matrix-BW = matrix backward; CFT 1–4 = subtests of the CFT 20-R. All paths were significant (*p* < .05).

In a next model, we investigated the predictive power of WM and age onto Gf. For this, we built a further model (Model 1b). Model 1b was computed equally as Model 1a, except that each Gf task was no longer regressed onto age. Instead, the Gf factor was regressed onto age to determine the direct and overall effect of age on Gf. In addition, Model 1b generated an excellent fit [*χ*^2^ (21) = 19.48, *p* = .55, normed *χ*^2^ = .93; CFI = 1.00; RMSEA = .00; SRMR = .04]. Together, WM and age explained 84% of the variance in Gf (see Figure A1 in Appendix A with further results of Model 1b). Because there was only one task per WM aspect, we were not able to further analyze the structure of WM [35].

In the next step, we built a model (Model 2a) testing the assumption that each WM aspect (and each task, respectively) uniquely contributes to the prediction of Gf. As mentioned above, simple and complex span tasks both measure storage, at least to some extent. Knowing this, several previous studies built nested models to test if processing still has a predictive value for Gf after controlling for storage. In these nested models, storage was controlled for by having all simple span tasks load on the processing factor [8,12,20,43,47,50]. However, none of the studies considered that in children, visual-spatial simple span tasks also demand processing [36,37]. Consequently, in the visual-spatial modality, common variance of simple span and complex span tasks seems to be mainly executive processing. As this proved to be the case in our data (see above), we built a model in which we regressed Gf onto the WM aspects while controlling for common variance among all WM tasks.

**Figure 3:**
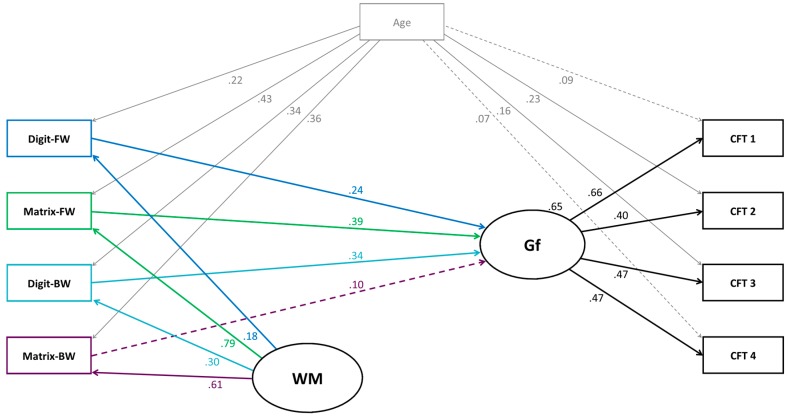
Structural equation model (Model 2a) testing the relations between working memory (WM) aspects and fluid intelligence (Gf). Age (in months) was included as control variable. Digit-FW = digit forward; Digit-BW = digit backward; Matrix-FW = matrix forward; Matrix-BW = matrix backward; CFT 1–4 = subtests of the CFT 20-R. *Solid lines* represent significant paths (*p* < .05), *dashed lines* represent non-significant paths (*p* > .05).

Model 2a was computed as Model 1a, except that Gf was not regressed onto the WM factor. Instead, Gf was separately regressed onto each WM aspect. See Figure 3 with the results of Model 2a. The model resulted in a very good fit and explained 42% of the variance in Gf [*χ*^2^(15) = 6.95, *p* = .96, normed *χ*^2^ = .46; CFI = 1.00; RMSEA = .00; SRMR = .02]. All regression coefficients in the model were significant at *p* < .05, except for three; namely, the coefficients from age onto two Gf subtests (CFT-1 and CFT-4), and from visual-spatial processing (respectively, matrix backward) onto Gf.

**Figure A2:**
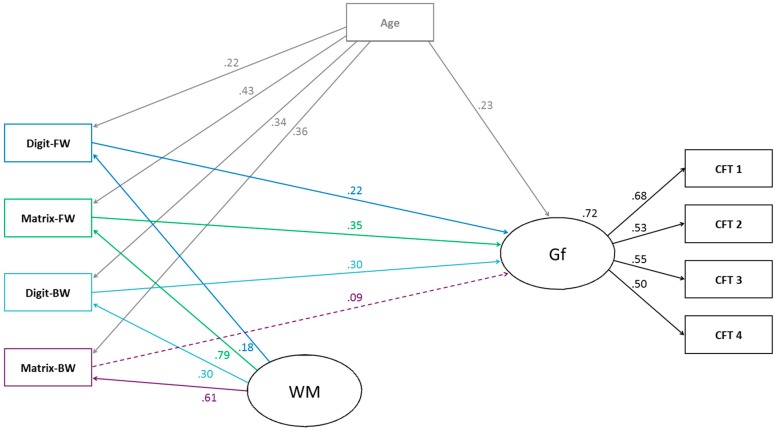
Structural equation model (Model 2b) testing the relations between working memory (WM) aspects, age and fluid intelligence (Gf). Digit-FW = digit forward; Digit-BW = digit backward; Matrix-FW = matrix forward; Matrix-BW = matrix backward; CFT 1–4 = subtests of the CFT 20-R. *Solid lines* represent significant paths (*p* < .05), *dashed lines* represent non-significant paths.

In the next model, we investigated the predictive power of each WM aspect and age onto Gf. This model (Model 2b) was similar to Model 2a, except that each Gf task was not regressed onto age. Instead the Gf factor was regressed onto age. Furthermore, Model 2b generated an excellent fit [*χ*^2^ (18) = 9.88, *p* = .94, normed *χ*^2^ = .55; CFI = 1.00; RMSEA = .00; SRMR = .03]. The WM aspects (after controlling their common variance) and age explained 51% of the variance in Gf (see Figure A2 in Appendix A with further results of Model 2b).

## 4. Discussion

In the present work, firstly, we investigated the inter-relations between simple and complex span tasks in elementary school children. Secondly, we explored the relation between one WM factor and Gf, and thirdly, we examined the relations between the different WM aspects (i.e., verbal storage, visual-spatial storage, verbal processing and visual-spatial processing) and Gf. In order to get “purer” estimations of the contribution of storage and processing, we analyzed the relations between WM aspects and Gf by controlling for the common variance among simple and complex span tasks. 

In short, the inter-relations revealed that—in general—simple and complex span tasks correlated positively and substantially, with a differential pattern across the two modalities: in the verbal modality, common variance seemed to mirror mainly storage. In contrast, in the visual-spatial modality, common variance seemed to mirror mainly executive processing. Loading all span tasks on one WM factor revealed a significant relation between the corresponding WM factor and Gf. However, when examining the unique contribution of the four WM aspects separately for Gf, results revealed that verbal storage, visual-spatial storage and verbal processing predicted unique variance in Gf. For visual-spatial processing, this was not the case. In the remainder of this discussion, we consider some limiting conditions on this evidence and discuss our findings in the context of prior work.

There are limits to drawing firm conclusions of our results. First, our data are not longitudinal. A longitudinal design would allow analyzing the directions of the relations between the WM aspects and Gf. In addition, development differences in the relations among the WM aspects and Gf could be explored. The latter issue would be important because our results indicate that age explains a substantial amount of variance in Gf. Furthermore, Demetriou et al. [67] showed that the strength of the relation between WM and Gf varies with age. They found that Gf is strongly linked to WM in the age ranges 9–11 and 14–16. In the age ranges of 6–8 and 11–13, in contrast, WM appears to be less closely linked to Gf. A second limitation of the present study is that we included only one task per concept. Consequently, we cannot draw very firm conclusions about the theoretical structure of the WM system. For this purpose, more tasks per latent variable would be necessary [35]. Therefore, we interpret our data more on the task- than on the construct-level. Future studies might want to investigate if our results generalize to other and to more tasks per WM aspect. For example, a previous study with older adults showed that backward span tasks are easier compared to other complex span tasks [68]. Thus, it might be good to include additional and more difficult complex span tasks. Having said that, however, the construction of very similar tasks (forward and backward span) in two distinct modalities in fact constitutes a strength of our approach. A third limitation of the present study is that WM is a multi-faceted construct that comprises several information processes (e.g., attention, inhibition, primary memory, secondary memory and speed of processing; [28,29,30,34]). With our selection and construction of tasks, we were not able to capture all these processes but aimed—for theoretical reasons—at focusing on the distinction between storage and processing as a first step. Future research will address the remaining open issues.

### 4.1. Inter-Relations between Simple and Complex Span Tasks

Because one of the aims of our approach was to better understand the inter-relations between the simple and complex span tasks used, we will discuss those results in more detail. The inter-relations between simple and complex span variables revealed significant correlations among each other including two exceptions (verbal simple span did not correlate with both visual-spatial spans). These otherwise substantial correlations among simple and complex spans indicate that the complex span tasks also demand storage. However, it is also possible that simple span tasks demand executive processing, at least as from a certain degree of complexity [37,69].

Furthermore, our data showed that simple and complex span tasks share a substantial amount of variance within each modality (within the verbal and visual-spatial modality, respectively). This is most likely due to a similar kind of representation of this information [70]. In line with this interpretation, the relation between the two complex span variables was much weaker, and the relation between the two simple span variables was not significant. This may point to different kinds of information representations in memory [70]. 

The substantial links between simple and complex span tasks within each modality raise the question if our simple and complex span tasks measured different concepts (storage and WM), or if they possibly tapped the same basic processes. On the one hand, Unsworth and Engle [71], for example, argue that simple and complex span tasks do not represent different concepts like short-term storage (respectively, short-term memory) and WM (storage and processing). They argue that these tasks differ only from each other in terms of the relative emphasis in which basis information processes are involved, at least in adults. On the other hand, other authors argue that storage and executive processing represent different concepts, at least in children [12,43,48,50]. Together, therefore, findings concerning the question of whether or not simple and complex span tasks measure different concepts are inconsistent. As for the present study, we did not include enough different tasks per concept to answer this question. However, we can state that inter-relations (within the modalities: verbal *r* = .47; visual-spatial *r* = .58) indicate simple and complex span tasks to share a substantial amount of variance within each modality. However, the magnitudes of the correlations still indicate that the two tasks are not identical.

A closer look at the visual-spatial simple span variable revealed that it correlates with both complex span tasks. The strength of these correlations was moderate to large (correlation with verbal complex span: *r* = .28; with visual-spatial complex span: *r* = .58), and therefore stronger than expected. This led us to conclude that, in our study, the visual-spatial simple span task also demanded processing. Consequently, this indicates that the shared variance between both visual-spatial variables mirrors a substantial amount of processing in addition to storage.

Our interpretation is in line with van der Ven et al. [72], who analyzed a giant database (Math Garden). They found that, within the visual-spatial modality, item difficulty was very similar when forward and backward span tasks were compared. They assume that visual-spatial simple span tasks require active executive processing. This assumption was confirmed by Ang and Lee [36,37], who directly analyzed the cognitive processes underlying spatial simple span tasks (Corsi Blocks and Visual Patterns Test) in children. They found that both simple span tasks demanded executive processes, yet to a different extent.

Taken together, results regarding the inter-relations indicate that simple and complex spans share common processes. This emphasizes the necessity to control for common variance between simple and complex spans, when the relation between WM aspects and Gf shall be analyzed.

### 4.2. Relationship between WM and Gf

As expected, mapping all span tasks on one general WM factor revealed a significant relation among the resulting WM factor and Gf. This finding is in line with several other studies showing that WM explains a large proportion of variance in Gf [11,73]. Including age into the model as a predictor of Gf resulted in even more variance of Gf being explained. However, when the four WM aspects were separately related to Gf, only three of the four WM aspects explained unique variance in Gf. The WM aspects that substantially contributed to the WM–intelligence link in children were verbal storage, visual-spatial storage and verbal processing. However, visual-spatial processing did not explain additional variance in Gf over and beyond the other WM aspects. It is noteworthy that the three WM aspects explaining variance in Gf had a comparable predictive power. These findings suggest that there is no predominance of any of the three tasks in the prediction of Gf, but rather that each of them contributes uniquely. This indicates that the different tasks trigger distinct information processes, all of which seem to be involved in Gf. In the next paragraphs, we will interpret these findings in the context of previous studies. First, we will consider the relations between verbal and visual-spatial storage and Gf. Second, we will discuss the relations between verbal and visual-spatial processing and Gf.

The finding that verbal and visual-spatial storage explained unique variance in Gf confirms results from Hornung et al. [50] as well as Tillman et al. [49]. These authors investigated storage separately for the verbal and visual-spatial modality and also found that verbal and visual-spatial storage to predict variance in Gf. At the same time, Gray et al. [52], who also investigated storage separately for both modalities, found that only visual-spatial storage was related to Gf, but not verbal storage. This underlines that visual-spatial storage involves processes shared with Gf (for similar findings, see: [6,7,48]). Therefore, our results, together with previous findings, indicate that visual-spatial storage is a predictor of Gf. 

The finding that only verbal processing but not visual-spatial processing explained unique variance in Gf is surprising. In particular because of the following two reasons: first, in the figural Gf test, children had to operate with visually overlapping features. This led to the expectation that better visual-spatial processing abilities would yield better results in the Gf test. Second, previous studies suggest that verbal as well as visual-spatial processing explain variance in Gf [33,43,49]. 

The result that verbal processing explained variance in Gf is in line with Tillman et al. [49] and other studies investigating processing only for the verbal modality [7,8,48,50]. However, the finding that visual-spatial processing did not explain variance in Gf contradicts previous findings [49]. Looking at the inter-relations among the four WM aspects included in the present approach provides one possible explanation why visual-spatial processing did not explain variance in Gf: the inter-relations indicate that in the visual-spatial modality, simple and complex span tasks trigger both storage and executive processing. Hence, it is feasible that visual-spatial processing could not explain additional variance in Gf over and beyond visual-spatial “storage”. Consequently, visual-spatial “storage” has to be interpreted with caution. We controlled for the common variance among simple and complex span tasks in order to get “purer” estimations of the contribution of storage and processing. However, it is not clear what variance is captured with this common WM factor, while we regressed Gf on each WM task. Was it storage or was it processing variance, or both? As all four WM tasks significantly loaded on this common factor, it is possible that common variance represented mainly storage [20]. Thus, the question arises if the variance left in visual-spatial “storage” actually represents more processing than storage variance. Therefore, even if the path from visual-spatial processing onto Gf was not significant, we cannot rule out the possibility that visual-spatial processing variance does not predict variance in Gf. The question of what represents the remaining variance after controlling for shared variance between WM span tasks is a general, yet open question in WM research, a question that has to be addressed in future research [30].

Taken together, our results indicate that individual differences in verbal storage, visual-spatial “storage” (or whatever the Matrix forward task measures) and verbal processing predict unique individual differences in Gf. However, future research needs to investigate if our findings are replicable with “purer” storage and processing tasks. 

## 5. Conclusions 

In the present study, inter-relations among simple and complex span tasks were examined. In addition, the relation between one general WM factor and Gf was analyzed. Lastly, relations between different, more circumscribed WM aspects and Gf were explored. Results concerning the inter-relations revealed that simple (forward) and complex (backward) span tasks share common processes. More precisely, the visual-spatial simple span task used also demands executive processes. Results concerning the relations between WM and Gf revealed the following: (a) loading all WM tasks on one factor explained substantial variance in Gf; (b) regressing Gf onto each WM aspect separately revealed that verbal and visual-spatial storage and verbal processing predicted unique variance in Gf, when holding the other effects constant. Thus, we are tempted to conclude that children who perform better in intelligence tests have better WM abilities such as better verbal storage, visual-spatial “storage” and more efficient verbal processing abilities.

## Figures and Tables

**Table 1:** Simple and complex span tasks used in the present study to assess the WM aspects.

Simple Span Tasks		Complex Span Tasks	
Verbal	Visual-Spatial	Verbal	Visual-Spatial
Digit Forward Task (Digit-FW)	Matrix Forward Task (Matrix-FW)	Digit Backward Task (Digit-BW)	Matrix Backward Task (Matrix-BW)
*Digit Recall task* from the Working Memory Test Battery for Children (WMTB-C; [55]).	Adapted version of the *Matrix subtest* from the Arbeitsgedächtnistest-batterie für Kinder von 5 bis 12 Jahren (AGTB 5–12; [56]).	*Backward Digit Recall task* from the WMTB-C [55].	Adapted version of the *Matrix subtest* from the AGTB 5–12 [56].

**Table 2:** Descriptive statistics of raw scores for all variables included in the study.

Variables	*Mean*	*SD*	Range	Skewness	Kurtosis
Digit Forward (verbal)	20.23	3.30	13–27	0.07	−0.67
Matrix Forward (visual-spatial)	15.65	4.11	3–24	−0.32	−0.43
Digit Backward (verbal)	13.98	3.69	6–24	0.21	−0.26
Matrix Backward (visual-spatial)	14.48	3.91	7–24	0.23	−0.24
Series Completion	10.09	2.31	3–14	−0.61	−0.20
Classification	7.26	2.23	2–13	0.09	−0.50
Matrix Completion	9.10	2.37	1–14	−0.66	0.28
Topological Reasoning	4.46	1.82	0–9	−0.05	−0.34
Age (in years)	10.63	1.02	9–12	−0.21	−1.66

**Table 3:** Pearson correlations and partial correlations controlling for age between span tasks and Gf.

Variables	Simple Span Tasks		Complex Span Tasks		Intelligence
	Digit-FW	Matrix-FW	Digit-BW	Matrix-BW	CFT Score
Digit Forward (Digit-FW)	-	.16	.47 ***	.12	.38 ***
Matrix Forward (Matrix-FW)	.24 **	-	.28 **	.58 ***	.43 ***
Digit Backward (Digit-BW)	.50 ***	.38 ***	-	.21 *	.44 ***
Matrix Backward (Matrix-BW)	.19 *	.64 ***	.31 ***	-	.30 **
Intelligence Composite Score (CFT Score)	.43 ***	.54 ***	.52 ***	.42 ***	-
Age (in years)	.22 *	.43 ***	.34 ***	.36 ***	.45 ***

Note: Pearson correlations below the diagonal; partial correlations controlling for age (in months) above the diagonal; * *p* < 0.5; ** *p* < 0.01; *** *p* < 0.001.
